# Design and Implementation of a Competency-Based Training Program for Specialty Pharmacists in China

**DOI:** 10.3390/pharmacy13060155

**Published:** 2025-11-01

**Authors:** Hamza El Alami, Ruoxin Huang, Nan Wu, Yufen Zheng, Pengyuan Wang

**Affiliations:** 1 School of Basic Medicine and Clinical Pharmacy, China Pharmaceutical University, Nanjing 210009, China; 3924090008@stu.cpu.edu.cn (H.E.A.); ruoxin23huang@163.com (R.H.); 2 Jiangsu Provincial Cancer Hospital, Nanjing 210009, China

**Keywords:** pharmacy education, competency-based education, case-based learning, OSCE, direct-to-patient pharmacy

## Abstract

This study describes the design, implementation, and evaluation of a Competency-Based Pharmacy Education (CBPE) program for 120 direct-to-patient (DTP) pharmacists in China, aimed at improving pharmaceutical care and pharmacotherapy skills. The program, which integrated Case-Based Learning (CBL) and Objective Structured Clinical Examination (OSCE), included both online and in-person sessions. A cross-sectional analysis of participant performance and satisfaction showed a mean total OSCE score of 68.31. Participants demonstrated strengths in communication and documentation, with one-third of participants achieving high scores, while weaknesses were noted in patient education and care planning. Participant surveys revealed significant perceived improvements in communication and patient education skills among 62.5% of the participants. These findings suggest that CBPE is a promising approach for pharmacist training, advocating for its broader adoption to meet the evolving demands of healthcare and improve patient outcomes.

## 1. Introduction

Community pharmacy practice is transitioning from a focus on medication preparation and dispensing to becoming an accessible healthcare destination providing care to patients when and where they need it [1]. Community pharmacists are expected to collaborate with other healthcare professionals and provide enhanced pharmacy services [2]. Specialty pharmacists, referred to as direct-to-patient (DTP) pharmacists in China, are a subset of community pharmacists who specialize in managing drugs for specific diseases requiring professional and personalized treatment services [3]. DTP pharmacists cater to patients by dispensing high-cost, high-touch medication, from oral to cutting-edge injectable and biologic products [4], offering specialized care that is both accessible and essential. The rapidly growing global cancer burden caused by population aging, growth, and increased exposure to risk factors [5], paired with the recent increase in oral agents for cancer [6,7], has caused DTP pharmacies to gain popularity, as they help patients and providers navigate financial and logistical barriers related to cancer treatment [8]. These trends place significant demands on DTP pharmacists to possess in-depth knowledge and refined clinical decision-making skills, as they are expected to provide patient counseling and education on adverse effects and self-management strategies, in addition to monitoring for medication toxicities and recommending dose adjustments as appropriate [9]. These changes present significant challenges to DTP pharmacists in China—a situation underscored by their current educational profile: 85.6% of licensed pharmacists have only completed a high school or associate program, with just 13.7% holding a bachelor’s degree, and only 0.7% possessing graduate credentials [10]. This inadequate composition of academic qualifications adds a significant strain to an already burdened healthcare system and complicates patients’ access to standard pharmaceutical care, emphasizing the urgent need for targeted professional development programs for DTP pharmacists. Simultaneous with paradigm shifts in pharmacy practice, recent shifts in pharmacy education have transitioned from traditional methods to competency-based pharmacy education, allowing innovative educational methodologies such as CBL and OSCE to be adopted and thrive in pharmacy education [11]. CBL involves both case discussions and simulations, promotes critical thinking by immersing trainees in realistic clinical scenarios [12], and has a long history and strong legacy of positive outcomes in medical and pharmacy education [13]. OSCE, on the other hand, provides a structured and standardized approach to evaluating skills through simulated clinical tasks [14]. OSCE usually consists of a circuit of several “stations” in loops testing the clinical competencies of examinees using a set of standardized checklists. All examinees rotate through the same set of stations simultaneously, perform identical defined tasks in the same time frame, are assessed by the same set of examiners, and are rated using pre-designed validated checklists by the examiners [15]. To address the challenges faced by DTP pharmacists, we formed an expert panel consisting of senior DTP pharmacists, DTP pharmacy managers, clinical pharmacists and faculty members to design and implement a CBPE-focused training program tailored to DTP pharmacists’ competency needs. The objective of this study was to evaluate the feasibility, acceptability, and initial outcomes of this program, with the goal of creating a replicable and scalable model for advancing pharmacy education in response to the growing demands of modern healthcare systems.

## 2. Materials and Methods

### 2.1. Study Design and Participants

This cross-sectional study targets DTP pharmacists in China. A nationwide call for applicants to our program was issued through professional DTP pharmacy channels across 24 provinces, ensuring a geographically diverse sample. The screening criteria for participants in our study were: (1) China-Licensed pharmacists (2) currently practicing in a DTP pharmacy. Non-DTP pharmacists were excluded from the study. All participants provided informed consent, and the project was approved by the Jiangsu Provincial Cancer Hospital Ethics Committee (Project ID: KY-2024-047).

### 2.2. Expert Panel

The expert panel consisted of DTP pharmacy managers, DTP senior pharmacists, clinical pharmacists, and faculty members. These experts were responsible for program conceptualization and implementation. Additionally, a task force was formed for CBL and OSCE design. The task force selection criteria were as follows: (1) licensed pharmacists holding a PharmD or PhD (2) extensive knowledge of cancer pharmacotherapy (3) a minimum of three years of pharmaceutical care training and (4) certification as instructors in pharmaceutical care practice.

### 2.3. Conceptualization

**Figure 1:**
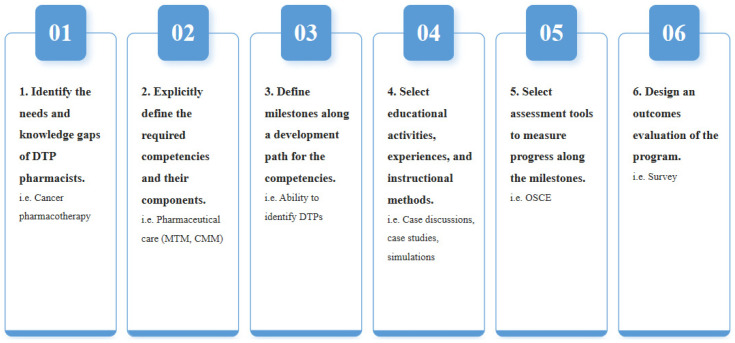
Curriculum Development Process.

Our CBPE curriculum followed a specific sequence adapted from the work of Frank et al. [16] as well as Kern’s six-step approach [17], as detailed in Figure 1. Recognizing the relatively low academic qualifications of DTP pharmacists in China, the curriculum focused on competencies in pharmaceutical care such as medication therapy management (MTM) and patient education, as well as pharmacology and cancer pharmacotherapy. Our main goal was to equip DTP pharmacists with the knowledge, skills, and confidence necessary to provide adequate pharmaceutical services and achieve optimal patient outcomes.

Figure 1 shows the stepwise conceptualization and design process of the Competency-Based Pharmacy Education (CBPE) program adapted from Frank et al. and Kern’s six-step approach with sequential stages from needs assessment to implementation and evaluation. DTP: Drug Therapy Problems, MTM: Medication Therapy Management, CMM: Comprehensive Medication Management, OSCE: Objective Structured Clinical Examination.

Additionally, study and simulation cases were developed from real-world scenarios. In order to align the content with participants’ knowledge levels, the material was iteratively refined based on experts’ feedback until consensus was reached. Case discussions and simulations aimed to enhance clinical decision-making skills within a patient-centered framework. Initial cases focused on single learning objectives and progressively evolved into complex, comprehensive scenarios to simulate realistic clinical situations, such as the example provided in Supplementary Materials. Following a case-based learning period, participants were assessed through OSCE. In the literature [18], opinions vary widely regarding the optimal number of OSCE stations. Since properly trained academic staff was limited, we opted for a four-station OSCE, each assessing specific competencies: (1) Case Preparation: Analyzing and synthesizing patient data, (2) Information Collection: Gathering clinically relevant information during patient interactions, (3) SOAP Documentation: Documenting subjective and objective patient information, assessing the patient’s overall health status, and creating an appropriate management plan, and (4) Patient Education: Delivering clear, concise, and personalized education on medication use, side effects, and adherence, as well as appropriate lifestyle changes. After completing the OSCE, participants were asked to complete a self-satisfaction survey.

### 2.4. Implementation

**Figure 2:**
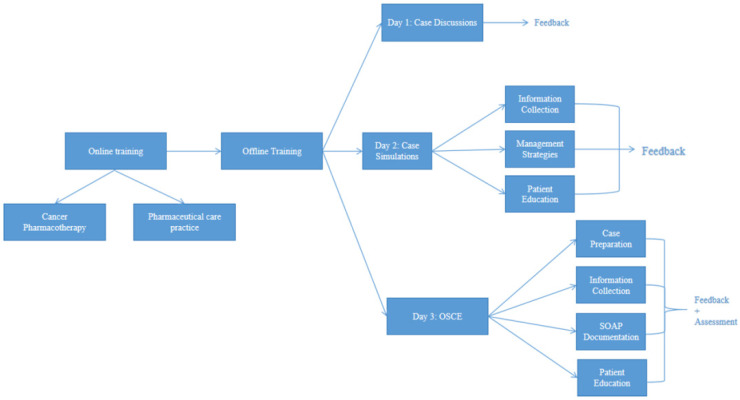
CBPE Training Implementation Process.

The training program was divided into online and in-person training, as shown in Figure 2. The online courses included 90 min weekly classes for 14 weeks, in addition to 30 min of out-of-class work, consisting of a patient case analysis, followed by a case discussion at the start of each week. These courses focused primarily on cancer pharmacotherapy and pharmaceutical care practice: Cancer pharmacotherapy courses offered an in-depth review of cancer immunopathology, in addition to cancer therapy, with a focus on chemotherapy, immunotherapy, and targeted treatments. Pharmaceutical care practice courses offered, on the other hand, an introduction to MTM, as well as patient education and interprofessional communication training. The in-person sessions spanned three days and consisted of case discussions, simulations as well as OSCE. For case simulations, participants were divided into groups and assigned a standardized patient (SP). The OSCE featured four interconnected stations: a first 10 min station involved retrieval of patient information and interview preparation. In the second station, participants interviewed SPs, with each interview lasting no longer than 10 min. Participants were expected to retrieve relevant patient information including but not limited to past medical history, chief complaint, medication and allergy history. During the third station, participants were given 30 min to write a SOAP note documenting the patient’s subjective and objective data, and providing the patient’s status assessment as well as a management plan. Participants would then move to a fourth station, where they had 10 min to provide patient education to their SPs. Finally, discipline-specific examiners evaluated each participant using standardized checklists.

Overview of the blended training structure combining 14 weeks of online learning with a 3-day in-person module, including case-based sessions and OSCE assessment. OSCE: Objective Structured Clinical Examination, SOAP: subjective, objective, assessment and plan.

### 2.5. Data Collection

Sociodemographic information included sex, age, and work experience was collected into our database. OSCE performance scores were collected onto separate Excel sheets and later analyzed. Data originally in Chinese was translated to English, and quality was assured with double translation.

#### 2.5.1. Performance Assessment

Participants’ performance was evaluated through OSCE. Each section was assessed using standardized checklists developed by the expert panel. The checklists included pre-validated criteria for scoring, ensuring consistency and objectivity. Performance scores from OSCE were recorded on a 1–5 scale for each criterion, with a total possible score of 100 per participant, then converted to a letter scale A (≥90), B (80–89), C (70–79), D (<70).

#### 2.5.2. Participant Perception Survey

The survey was divided into two parts: A Likert scale with five possible responses was utilized to assess participants’ perceived skills improvement using CBPE, rating ranged from 1 (no improvement) to 5 (significant improvement). Reliability of the Likert Scale was assessed by calculating Cronbach’s α coefficient. Multiple-choice questions were used to assess the other items.

#### 2.5.3. Statistical Analysis

Data were first downloaded to an Excel spreadsheet, and imported into IBM SPSS (version 27.0) for descriptive and inferential statistics. Performance Data: Mean scores for OSCE were tabulated, highlighting the competencies where participants excelled or required improvement. Survey Responses: Likert-scale results were summarized as mean scores, standard deviations and percentages. Multiple-choice questions were categorized thematically to identify recurring strengths and challenges. A Chi-Square test of independence was performed to examine the association between prior MTM training and participants’ willingness to recommend our CBPE program (significance at *p* < 0.05).

## 3. Results

### 3.1. Participants

A total of 120 licensed DTP pharmacists were selected to participate in our study. Detailed demographics are provided in Table 1.

### 3.2. Case Simulations and OSCE Performance

**Figure 3:**
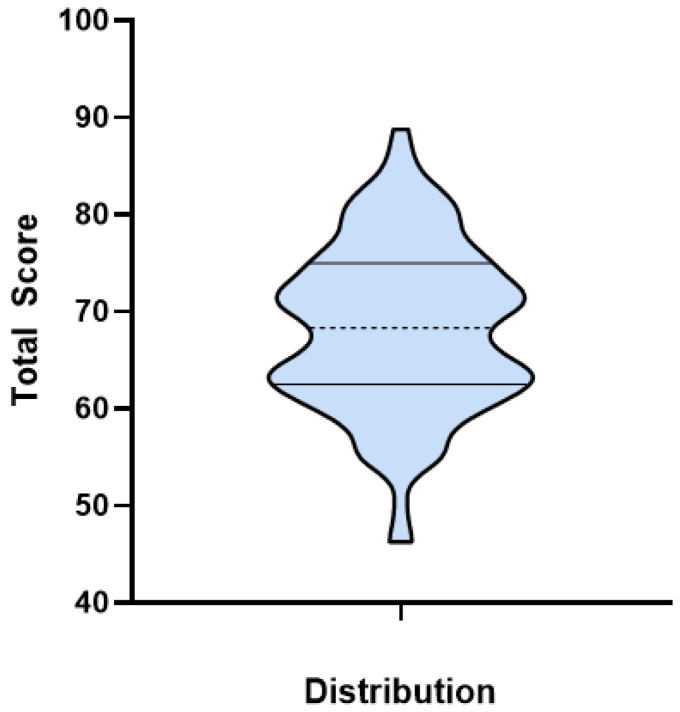
Participants Performance Total Score Distribution.

Overall, the performance mean total score was 68.31, and most of the participants performance scores fell within the 60–70 and 70–80 ranges, as shown in Figure 3, each accounting for 37.5% (*n* = 45).

**Figure 4:**
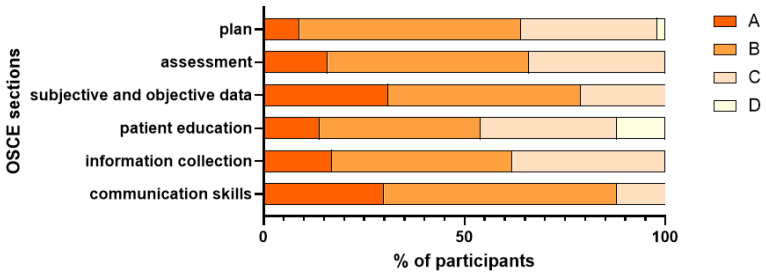
Participants’ Performance Distribution A: ≥90, B: 80–89, C: 70–79, D: <70.

Generally, participants performed best in the «communication skills» and «Subjective and Objective» documentation of the SOAP note, while their performance was relatively poor in patient education and plan, as shown in Figure 4.

### 3.3. Survey Findings

All participants (*n* = 120) completed the questionnaire. We performed an internal consistency test for the Likert-scale Survey, which showed a Cronbach’s α coefficient of 0.93, reflecting excellent internal consistency. Participants rated their skill improvements across various domains, with mean scores exceeding 4.2 on a 5-point Likert scale, as detailed in Table 2. Communication skills (4.48) and patient education ability (4.47) were rated highest, followed by teamwork ability (4.43) and clinical problem-solving ability (4.21).

The questionnaire also contained post-training survey questions regarding educational activities, perceived confidence, frequency of skill application, implementation barriers, and willingness to recommend the CBPE model, as summarized in Table 3.

Finally, our analysis showed no statistically significant association between previous MTM training and willingness to recommend our program (chi-square test, *p* = 0.4839). This indicates that the proportion of participants willing to recommend the program did not significantly differ based on their previous MTM training status.

## 4. Discussion

This program aimed to equip DTP pharmacists with the necessary skills to handle complex medical cases and patients prescribed with specialty drugs using CBL and OSCE as the main learning tools. Following the program completion, a descriptive cross-sectional study was performed, and a post-training satisfaction survey to evaluate both performance outcomes and perceived improvements in various competencies was completed.

### 4.1. CBL and OSCE

Participants’ training incorporated CBL as a foundational approach, emphasizing the application of theoretical knowledge to practical, patient-centered scenarios. Previous findings from Gui et al. [19] showed the superiority of CBL to traditional teaching approaches. Hence, CBL provided a strong preparatory framework for participants’ subsequent OSCE.

During OSCE, the scenario we used was comprehensive and multifaceted. Participants were required to take patient history, identify multiple drug therapy problems, document interventions, and educate the patient. We believe this approach provides a sense of realism as participants are expected to deal with multiple tasks at the same time, just as they are expected to in their day-to-day practice.

Overall, participants demonstrated moderate performance in the OSCE. Performance scores highlighted strengths in communication skills and subjective and objective documentation. This aligns with the essential skills required for pharmacists [20], where clear patient communication and accurate documentation are essential for optimal pharmaceutical care. Despite these positive outcomes, certain limitations were evident in the results. A considerable proportion of participants scored lower in patient education competencies, suggesting a need for greater emphasis on this aspect during training. Patient education has been defined as a ‘planned learning experience, aimed to improve the knowledge, skills and health behavior of the patient’ [21]. Patient education involves not only the delivery of information but also the ability to assess patient understanding and promote adherence, which may require additional practical simulations and focused feedback. Participants’ performance scores in the assessment and plan documentation were also lower, indicating a potential area for further development in our curriculum.

### 4.2. Post-Training Self-Satisfaction Questionnaire

Survey responses further support the positive impact of the CBPE curriculum. Participants consistently rated their skill improvements highly, especially in communication skills, patient education, and teamwork. These findings suggest that the CBPE model, which emphasizes active learning and real-world simulation, may enhance practical, patient-centered skills. These results are in line with the findings of previous research [22,23,24,25,26], which reported increased self-assessment scores for various pharmaceutical care competencies following the adoption of CBL and OSCEs in pharmacy education. Among the teaching methods evaluated in our program, medication therapy management case discussion was rated as the most impactful, with 95% of participants affirming its utility in enhancing their professional competencies. This is closely followed by case simulations (75.83%) and OSCE (75%).

Furthermore, our analysis revealed that participants’ willingness to recommend the CBPE training was not significantly associated with their prior MTM experience (*p* = 0.4839). This non-significant finding is noteworthy, as it suggests that the CBPE model is well-received by DTP pharmacists across different levels of prior professional exposure. When this finding is considered alongside the high overall recommendation rate (91.66% were willing/very willing), it may indicate the program’s broad applicability, suggesting its design could be beneficial for pharmacists with both limited and extensive MTM background.

Another interesting finding was the difference between theoretical knowledge and real-world practice, as highlighted by participant feedback. While CBL and OSCE prepare pharmacists to face real patients to some extent, the variability and complexity of real-world cases often surpass the controlled scenarios used in training. Moreover, the weeks leading up to an OSCE are generally filled with immense preparation on the participants’ behalf, showcasing the maximum performance competency level, as opposed to the reality of real-life practice [27], where work conditions are often suboptimal and pharmacists are required to balance between multiple professional tasks. This gap may be addressed by incorporating more diverse case simulations and multidisciplinary collaboration within the training modules, with gradually increasing difficulty, which could further enrich the learning experience and better prepare pharmacists for complex clinical environments. Additionally, challenges related to time management were frequently reported by participants. Balancing medication therapy management responsibilities with other professional duties remains a prevalent issue. This suggests the need for targeted modules on workflow optimization and time management strategies as part of future training iterations. Other challenges reported were lack of organizational and structural support, which was also mentioned by Katoue et al. [28], who emphasized that the successful implementation of competency-based education tools requires strong and supportive institutional leadership.

### 4.3. Pharmacist Continued Education Through CBPE

Continued education for pharmacists is essential for maintaining and improving their competencies throughout their careers [29], particularly in an ever-evolving healthcare landscape, and has become a requirement for license renewal for board certified pharmacists in some countries [30]. Pharmacists must stay abreast of emerging drug therapies, evolving clinical guidelines, and advancements in pharmacology to provide the highest level of patient care. CBPE serves as a structured and effective model for addressing these needs by ensuring that pharmacists acquire the specific skills and knowledge required to meet current healthcare demands.

Continued education through CBPE emphasizes not only theoretical learning but also practical application through case simulations and assessments, ensuring that pharmacists can translate knowledge into action in real-world settings. This is especially relevant with the paradigm shift in pharmacy practice, from medication providers to clinical practitioners. This approach enables pharmacists to build confidence in their ability to address complex drug therapy problems, improve patient outcomes, and contribute to multidisciplinary teams through collaborative decision-making. Training through CBPE ensures that pharmacists are well-equipped to meet these evolving responsibilities and provide optimal care throughout their careers.

### 4.4. Barriers to Implementation of a CBPE Curriculum

Despite its benefits, the implementation of CBPE is not without its challenges. One significant barrier is the need for strong institutional support and leadership. In our study, the success of the program was contingent on the active involvement of experts and faculty members, but broader institutional commitment was necessary to sustain the model and overcome resistance to change. This is also supported by Sibicky et al. [31], who found that CBPE drivers included developing support systems for stakeholders, shifting the organizational culture away from learner differentiation toward competence, and maintaining sufficient administrative capability to support CBPE. Additionally, faculty development is crucial for the successful adoption of CBPE, as instructors must be trained in formative assessment and feedback techniques specific to competency-based learning. Furthermore, the integration of educational technologies is essential for tracking competencies and enhancing the learning experience. However, logistical challenges such as time constraints and the need for advanced digital tools to support the CBPE process must be addressed to ensure scalability and sustainability. Overcoming these barriers will require a concerted effort from all stakeholders, including administrators, faculty, and policymakers, to create an environment that supports the effective implementation of CBPE.

### 4.5. Limitations

It is important to contextualize the generalizability of our findings within the constraints of the study’s recruitment. The *n* = 120 participant size was determined primarily by operational capacity (instructor availability and resource constraints) rather than a statistical power calculation against the total national population of DTP pharmacists, since to our knowledge, there is no study or official report providing that total population in China. Furthermore, the nationwide call was executed through targeted professional networks, resulting in a convenience sample of professionals who were both interested in and able to commit to specialized training. Consequently, our findings reflect the initial outcomes and acceptability among a highly motivated subset of DTP pharmacists, and may not be broadly generalizable to the entire Chinese DTP pharmacist population or to other geographical regions.

Additionally, the cross-sectional, post-intervention design, which lacked a baseline assessment and a suitable control group, precludes us from establishing a causal link between the CBPE intervention and the observed performance or satisfaction outcomes. The demonstrated skills may reflect a combination of the training’s effectiveness and the inherent high level of professional commitment and skill of the pharmacists who self-selected to enroll in a specialized CBPE course. Follow-up studies with a longitudinal design would offer a more comprehensive understanding of the long-term impact of the CBPE curriculum.

Furthermore, due to the limited number of instructors, our OSCE consisted of only four stations, which may have constrained the scope of competency assessment. Also, the use of an online questionnaire to collect data creates both a response bias and a recall bias. Finally, we acknowledge that others may disagree with the philosophical foundations of this work and have different interpretations of key educational concepts, particularly regarding the necessary scope of CBL and the psychometric requirements for OSCE. We therefore urge readers to conceptualize our findings within this pragmatic framework and its defined methodological assumptions.

## 5. Conclusions

In conclusion, this study provides evidence supporting the combined use of CBL and OSCE as a feasible model for targeted DTP pharmacist competency-based professional development and offers a reliable methodology for the potential integration of CBPE in the pharmacy education continuum. By addressing the identified limitations and continually refining the educational approach, a wide-scale implementation of CBPE training programs could play a pivotal role in enhancing the quality of pharmaceutical care in community settings. Future studies should explore longitudinal assessments to determine the sustained impact of these training methods on professional practice and patient outcomes.

## Figures and Tables

**Table 1:** Participants’ demographics.

Characteristics	*n*	(%)
Sex		
male	14	(11.7)
female	106	(88.3)
Age group		
26~30	2	(1.7)
31~40	63	(52.5)
41~50	49	(40.8)
51~60	6	(5.0)
Work experience		
0~5 years	5	(4.2)
6~10 years	24	(20.0)
11~20 years	63	(52.5)
20+ years	28	(23.3 )
Previous MTM experience		
Yes	78	(65.0)
No	42	(35.0)

MTM: Medication Therapy Management.

**Table 2:** Perceived skills improvements using CBPE as an education method.

Skill	1	2	3	4	5	Mean ± SD
Communication skills	2 (1.67%)	0 (0%)	11 (9.17%)	32 (26.67%)	75 (62.5%)	4.480 ± 0.799
Ability to design drug treatment plans	2 (1.67%)	4 (3.33%)	17 (14.17%)	30 (25%)	67 (55.83%)	4.300 ± 0.949
Patient Education ability	2 (1.67%)	2 (1.67%)	9 (7.5%)	32 (26.67%)	75 (62.5%)	4.470 ± 0.840
Clinical problem-solving skills	3 (2.5%)	2 (1.67%)	21 (17.5%)	35 (29.17%)	59 (49.17%)	4.210 ± 0.961
Teamwork ability	2 (1.67%)	0 (0%)	13 (10.83%)	35 (29.17%)	70 (58.33%)	4.430 ± 0.816

Likert scale, 1: No improvement, 5: Significant improvement.

**Table 3:** Summary of responses to post-training survey questions regarding educational activities, perceived confidence, frequency of skill application, implementation barriers, and willingness to recommend the CBPE model.

Question	Response Options	*n* (%)
Education activities that enhanced pharmaceutical service skills	Medication therapy management case discussion	114 (95%)
	Case simulation	91 (75.83%)
	OSCE	90 (75%)
	Group Discussion	74 (61.67%)
	Others	8 (6.67%)
Confidence in pharmaceutical service provision improved through CBPE	Significantly improved	76 (63.33)
	Partially improved	43 (35.83%)
	Unsure	1 (0.83%)
	No change	0
Frequency of applying learned skills in actual work	Frequently applied	74 (61.67%)
	Occasionally applied	40 (33.33%)
	Rarely applied	6 (5%)
	Never applied	0
Main difficulties encountered in applying learned skills	Difficulties with time management	86 (71.67%)
	Discrepancies between real-world medication therapy issues and simulated scenarios	81 (67.50%)
	Lack of structural and organizational support	44 (36.67%)
	Others	6 (5%)
Perception on whether CBPE can improve patient treatment outcomes	Very helpful	52 (43.33%)
	Somewhat helpful	64 (53.33%)
	The effect is not obvious	4 (3.33%)
	Not helpful	0
Suggested improvements for CBPE	Increase multidisciplinary cooperation content	97 (80.83%)
	Provide more practical opportunities	92 (76.67%)
	Increase the complexity of clinical scenarios	70 (58.33%)
	Strengthen feedback sessions	60 (50%)
	Others	4 (3.33%)
Willingness to recommend CBPE as a training method	Very willing	52 (43.33%)
	Willing	58 (48.33%)
	Unsure	9 (7.5%)
	Unwilling	1 (0.83%)

## Data Availability

The data presented in this study are available on request from the corresponding author due to privacy.

## Supplementary Materials

The following supporting information can be downloaded at: https://www.mdpi.com/article/10.3390/pharmacy13060155/s1, File S1: example of a study case.

## Abbreviations

The following abbreviations are used in this manuscript:

Abbreviation	Definition
CBL	Case-based learning
OSCE	Objective Structured Clinical Examination
DTP	Direct-to-patient
CBPE	Competency-based pharmacy education
MTM	Medication Therapy Management
SOAP	Subjective, objective, assessment, plan
SP	Standardized Patient
CMM	Comprehensive medication management
