# Supplementary material for: Design and Implementation of a Competency-Based Training Program for Specialty Pharmacists in China
Source: Pharmacy (Basel). 2025 Nov 1;13(6):155. doi: 10.3390/pharmacy13060155 (PMC12641960; doi:10.3390/pharmacy13060155)
Supplement: Supplementary file 1 [file pharmacy-13-00155-s001.zip › pharmacy-3890210-Supplementary Material File S1.pdf]

The First Assessment Breast Cancer Service Case 1

Basic Information: XXX, 56 years old, female, retired, divorced, 168cm, 58Kg, urban and rural resident medical insurance
Opening remarks: Hello, I have been a bit constipated lately. Is this related to my medication?
Chief complaint: Constipation accompanied by anorexia and fatigue for over 1 month.
History of present illness: Eight months ago, due to self-perceived breast distension and pain, she sought medical treatment and was diagnosed with breast cancer. She underwent surgery 6 months ago (right breast total mastectomy, right axillary lymph node dissection). Post-surgery: axillary lymph node metastasis (4/12), immunohistochemistry showed ER (+80%), PR (-), HER2 (-), Ki (+, 40%). Post-surgery, she received EC-T (Epirubicin, Cyclophosphamide, Docetaxel) chemotherapy. Two months ago, after completing the last cycle of chemotherapy, the doctor prescribed letrozole + abemaciclib for her to take. At the same time, due to osteoporosis concerns, calcium tablets and Vit D were prescribed for her to use. Constipation began to appear in the past month.

Questions:
If you want to provide a complete MTM service for this patient, what other information do you need to collect?

Based on the information you have collected, what drug-related problems does the patient have?

Based on the drug-related problems you have comprehensively assessed, what treatment plan will you formulate for the patient?

How will you work with the patient to implement the above treatment plan?

For this patient, how will you complete the follow-up assessment?

N.B: The content above was presented to the participants. The content below was the one used by the examiners and standardized patients to simulate the patient case and assess each participant's performance.

Information Collection

For the chief complaint—constipation: specific symptoms; presence of accompanying symptoms; relevant past medical history; whether self-treatment measures have been taken.

Diseases: other diseases and medications.

Medication: specific medication taken by the patient, dosage, usage, allergies, and side effects experienced; medication experience and expectations.

Other (additional) medication and treatment needs.

The complete case information is as follows:

XXX, 56 years old, female, retired worker, divorced, 168cm, 58Kg, urban and rural resident medical insurance

Opening remarks: Hello, I have been a bit constipated lately. Is this related to my medication?
Chief complaint: Constipation accompanied by anorexia and fatigue for over 1 month
History of present illness: 8 months ago, due to self-perceived breast distension and pain, she sought medical treatment and was diagnosed. Half a year ago, she underwent surgery (right breast total mastectomy, right axillary lymph node dissection). Post-surgery: axillary lymph node metastasis (4/12), immunohistochemistry showed ER (+80%), PR (-), HER2 (-), Ki (+, 40%). Post-surgery, she received EC-T (Epirubicin, Cyclophosphamide, Docetaxel) chemotherapy. Two months ago, after completing the last cycle of chemotherapy, the doctor prescribed letrozole + abemaciclib for her to take. At the same time, to prevent bone metabolism problems, calcium tablets and VD were prescribed for her to use. In the past month, she has gradually felt some anorexia and constipation, manifested as dry stools, difficult and time-consuming bowel movements, and a feeling of incomplete emptying. The stool color is dark brownish-yellow, and she has a bowel movement every 2-3 days. She has tried taking domperidone and drinking more water on her own to regulate it, but the effect has been poor, and the constipation has persisted. She wants to take some other medication for treatment. Recently, the patient reported some facial hot flashes, sweating, and tenderness on the back of the left calf.
Past medical history: No history of intestinal obstruction, no other chronic diseases.
Family and personal history: No special family history, menopause at 51 years old.
Lifestyle history: Lives with her husband, son, and daughter-in-law. The patient has had low energy for the past month, and coupled with the cold winter, she rarely goes out for activities and spends most of her time lying on the bed watching TV.
Allergy history: None.
Smoking and alcohol history: None.
Exercise: Little to no physical activity, spends a lot of time in bed recently.
Diet: Poor appetite for the past month, salt intake not calculated, prefers spicy food, no special dietary composition.

Vaccination: None

Medication experience: Good medication adherence. Recently, constipation problems have occasionally appeared, and she wonders if it is because of taking letrozole.

Medication expectations: To first receive medication to cure the constipation, and then hopes to receive some medication to improve her spirit.

Checkup report from 2 months ago (biochemistry):

The following table:

Item	Value	Reference range	Item	Value	Reference range
ALT	52.9U/L	9-50	AST	30.2 U/L	15-40
ALP	85 U/L	30-120	γ -GGT	20.3 U/L	10-60
TBIL	21.2umol/L	5-20	DBIL	5.6 umol/L	0-6.8
IBIL	17.8umol/L	0-20	HDL-C mmol/L	1.25	1.03-1.55
LDL-C	2.86mmol/L	2.60-4.10	TC mmol/L	4.68	3.00-5.70
TG	1.18mmol/L	0.00-2.25	GLU	6.26mmol/L	3.90-6.10
Urea	3.78mmol/L	2.90-8.20	Creatinine Cr	98.8umol/L	44.0-133.0
UA	329umol/L	208-428	Calcium Ca	2.45mmol/L	2.25-2.75
Phosphate P	0.79mmol/L	0.81-1.45	Magnesium Mg	0.81mmol/L	0.73-1.06
Potassium K	3.97mmol/L	3.50-5.50	Sodium Na	138.6mmol/L	137.0-147.0
Chloride Cl	106.2mmol/L	100.0-110.0	CysC	1.16mg/L	0.03-1.25
25-OH-VD	49ug/L	30-100ug/L			

BMD (Bone mineral density test): Lumbar spine (T4~T8) T-score -1.3, femoral neck T-score -1.3

Medication usage

Drug	Dosage	Frequency	Indication	Duration
Calcium carbonate D3 chewable tablets (Caltrate)	300mg：60IU	1 tablet, BID	Bone loss	2 months
Calcitriol soft capsules (Rocaltrol)	0.25ug	1 capsule, BID	Bone loss	2 months
Letrozole tablets (Femara)	2.5mg	1 tablet, QD	Breast cancer	2 months

Abemaciclib tablets (Verzenio)	150mg	1 tablet, QD	Breast cancer	2 months
Domperidone tablets (Motilium)	10mg	1 tablet, BID	Indigestion	2 months

Drug therapy problems:

Drug/Disease	Drug Therapy Problem	Actual/Potential	Risk	Rationale
Calcium carbonate D3 chewable tablets	Safety/Adherence	Actual	High	The doctor prescribed 1 tablet per day, but the patient self-increased the dose to 2 tablets per day, leading to excessive calcium supplementation and causing constipation.
Constipation	Indication	Actual	High	Lack of medication for treating constipation.
Letrozole	Safety	Actual	High	The patient developed menopausal-like adverse reactions such as hot flashes and sweating after taking letrozole.
Domperidone	Indication/Safety	Actual	High	1. Domperidone is not used for constipation treatment. 2. Domperidone is contraindicated in breast cancer patients. 3. Both abemaciclib and domperidone have the risk of prolonging the Q-T interval.
Venous thrombosis	Safety	Potential	Medium	The patient has local tenderness on the back of the left calf, and has been spending a lot of time

				in bed recently. At the same time, she is taking abemaciclib and letrozole, so venous thrombosis needs to be ruled out.
Calcitriol soft capsules	Indication	Actual	Medium	Blood 25-OH-VD > 30 ug/L, unnecessary drug therapy

Monitoring plan

Drug treatment goals: Treatment goals can include curing the disease, reducing or eliminating signs and/or symptoms, slowing or stopping the progression of the disease, preventing disease, and normalizing laboratory tests.

Alleviate constipation and menopausal syndrome symptoms.

Prevent the occurrence of adverse cardiac events such as prolonged Q-T interval.

Screen for bone marrow suppression and venous thrombosis, and prevent disease occurrence.

Non-drug interventions

-Constipation, menopausal syndrome: Advise the patient to consume enough dietary fiber, eat more vegetables, fruits, and bowel-lubricating foods, such as bananas, sweet potatoes, sesame oil, honey, etc. Avoid spicy, irritating, or "heat-producing" foods. Develop a habit of drinking water regularly and proactively, with a daily water intake of 1500-1700ml, 50-100ml each time, and drink warm boiled water or light tea. Develop a regular bowel movement habit; it is recommended to try to have a bowel movement in the morning or within 2 hours after a meal. Practice appropriate abdominal exercises to increase intestinal peristalsis; Practice physical activity, such as aerobics, yoga, tai chi, etc., and try to maintain an active lifestyle.

-Bone loss: Regular moderate-intensity exercise, eating calcium-rich foods, preventing falls and violent body impacts.

Drug interventions

Calcium carbonate D3 chewable tablets, Calcitriol soft capsules: Reduce the dose of calcium carbonate VD3 chewable tablets to 1 tablet QD, and advise the patient to stop using calcitriol capsules.

Domperidone: Stop using domperidone.

Constipation: Recommend lactulose oral solution (30ml once a day).

Venous thrombosis: Advise the patient to be admitted to the hospital to assess the risk of venous thrombosis.

Follow-up assessment:

Short-term follow-up (3-7 days): Improvement in constipation, improvement in menopausal syndrome, referral status.

Long-term follow-up : Adverse reactions of calcium carbonate D3 chewable tablets (constipation, hypercalcemia); adverse reactions of letrozole (arthralgia, osteoporosis, and other muscle and connective tissue abnormalities; hot flashes, sweating, and other menopausal-like adverse reactions; prolonged Q-T interval; thromboembolic events; lipid metabolism; headache, dizziness, and other nervous system symptoms; gastrointestinal; weight); adverse reactions of abemaciclib (bone marrow suppression, diarrhea, abnormal liver function, rash, prolonged Q-T interval, venous thromboembolism, interstitial pneumonia, etc.), tumors (tumor control).
